# An Approach to Collecting School District Level COVID-19 Mask Mandate Information in the United States form the Web using Tools Powered by Artificial Intelligence.

**DOI:** 10.23889/ijpds.v7i3.2016

**Published:** 2022-08-25

**Authors:** Sadaf Asrar, Imer Arnautovic, Dan Loew

**Affiliations:** 1 Optimal Solutions Group, LLC

The objective of this study was to collect online information about COVID-19 mask mandates in United States (U.S.) school districts using artificial intelligence to verify and contribute to official statistics collected by the U.S. Department of Education (ED) and linking to data reported by U.S. states.

To collect the data, the authors developed a customized web scraping tool using the Python package BeautifulSoup to automate Google searches of mask mandates in school districts in the U.S. These automated searches retrieved a pre-determined number of top search results in a tabular format. Next, the authors developed Natural Language Processing (NLP) code that read the search results and classified which school districts had implemented a mask mandate. This classification was achieved by developing and training a supervised machine learning algorithm using the search results data that were manually labelled by the authors.

As a pilot study, the authors were able to successfully develop the automated Google search tool to query and retrieve school district level mask mandate information for the state of Ohio. The algorithm trained using this data classified which school districts had implemented a mask mandate with an accuracy of 87 percent. The data predicted by the algorithm was used to verify the same data collected by ED through monthly surveys of public schools, and will inform masking policy data for states that had low response rates to the ED survey. These data will also be linked to data on the number of COVID-19 cases in schools reported by state governments to assess the success of masking policies in reducing COVID-19 case counts.

The results of the study demonstrate that large scale data collection and validation activities can be conducted with high accuracy at a low cost and can be repeated more frequently than surveys can without incurring any additional burden on potential respondents.

